# Local, non-systemic, and minimally invasive therapies for calcinosis cutis: a systematic review

**DOI:** 10.1007/s00403-021-02264-5

**Published:** 2021-06-24

**Authors:** Joanna Nowaczyk, Michał Zawistowski, Piotr Fiedor

**Affiliations:** Department of General and Transplantation Surgery, Medical University of Warsaw, Nowogrodzka 59, 02-006 Warsaw, Poland

**Keywords:** Calcinosis cutis, Calcifications, Extracorporeal shockwave lithotripsy, Laser, Sodium thiosulfate, Minimally invasive surgical procedures

## Abstract

Calcinosis cutis is a deposition of calcium in the skin and subcutaneous tissue, often accompanied by pain, reduced mobility, and chronic infections. Limited evidence is available about the feasibility and efficacy of therapies alternative to systemic treatment and surgical excision, both of which often lead to unsatisfactory results or complications. We conducted a systematic review to evaluate the efficacy and safety of topical and intralesional sodium thiosulfate, extracorporeal shock-wave lithotripsy (ESWL), and laser for calcinosis cutis. PubMed, Embase, and Web of Science were searched. Reports of calciphylaxis and treatment combined with systemic medications were excluded. A total of 40 studies including 136 patients were analysed. Partial or complete remission after monotherapy was observed in 64% to 81% of cases. Self-applied topical sodium thiosulfate required patient’s adherence (mean treatment duration, 4.9 months; range 2–24). Laser therapy enabled complete remission of microcalcifications after a single procedure (57%; 12/21). ESWL and intralesional sodium thiosulfate injections decreased calcinosis-associated pain (median reduction in VAS score, 3; range 0–9 and 1; range 0–5, respectively). The most common adverse event was scarring and hyperkeratosis, observed after CO_2_ laser (56%; 10/18). Intralesional sodium thiosulfate injections caused transient pain in over 11% of patients. Recurrences within the follow-up were rare (2%; 3/136). This study provides an overview of minimally invasive and local therapies that in selected cases might transcend conventional treatment. The limitation of this study is the poor level of evidence, which emerges mainly from non-randomized studies at high risk of bias.

**Supplementary Information:**

The online version contains supplementary material available at 10.1007/s00403-021-02264-5.

## Introduction

Calcinosis cutis (CC) is a cutaneous and subcutaneous deposition of insoluble salts of calcium. Depending on the underlying cause, calcifications are classified into four types: dystrophic (e.g. caused by trauma or connective tissue disease), metastatic (e.g. in chronic kidney disease), iatrogenic (e.g. after intravenous fluids extravasation), and idiopathic (formed without any apparent reason) [1]. A severe course of disease is often associated with pain, reduced range of motion, ulcerations, cosmetic concerns, and recurrent infections [2, 3], that may even result in extremity amputation [4] and mental health problems. Poor quality of life and significant disability are the main concerns of patients with advanced CC.

Based on limited evidence about numerous therapies reported, definitive treatment recommendations for CC have not been established [3, 5, 6]. Data regarding the efficacy of several off-label oral and intravenous drugs are inconsistent [7, 8] and their use is limited by such factors as: severity of the underlying disease, drug interactions, increased risk of severe adverse events, and the need for institutional review board approval. In these situations, treatment applied locally may be more suitable due to a minimal systemic influence [9]. Surgical extraction is often successful, nonetheless carries the risk of complications due to tissue injury, yet recurrence of the lesion [6, 7]. Excision of large lesions may require muscle amputation [4, 10] and skin grafting [11, 12], while surgical removal of fingertip calcifications may cause digital neurovascular bundle damage and limited mobility [2, 7, 12–14].

In this systematic review, we summarised the current knowledge about minimally invasive and local treatments for CC, including laser therapy, extracorporeal shockwave lithotripsy (ESWL), topical (top-STS) and intralesional sodium thiosulfate (IL-STS).

## Materials and methods

### Protocol and registration

This study is based on a prospectively written protocol that has been registered on PROSPERO (CRD42020207253). Results are reported in compliance with the PRISMA statement [15].

### Search strategy

We searched PubMed, Embase, and Web of Science for eligible studies. The search was complemented by the manual screening of papers’ references. Additional search information is summarised in the Supplementary Content.

### Eligibility criteria

Studies reporting the use of top-STS, IL-STS, ESWL, and all kinds of laser therapies for CC were considered eligible for inclusion. Full-text articles as well as conference abstracts of acceptable quality in English, Spanish, or German were evaluated. Studies reporting patients with calciphylaxis as well as individuals treated with a combination of systemic and local or minimally invasive treatment were excluded at the screening stage.

### Data extraction

Two reviewers independently screened the records yielded by the search. Discrepancies were resolved by discussion among all co-authors. The following variables were extracted from the articles: type of study, number of patients, their age, sex, main and additional diagnoses, location and size of the lesions, duration of the symptoms, additional features of the lesions (e.g. ulceration), type of therapy used for calcinosis cutis (with dosage, regimen, and route of administration), follow-up, outcomes of the therapy, adverse effects, previous treatment used for calcinosis cutis, and the underlying cause of calcinosis. Additionally, we extracted any available information about indications and contraindications of the evaluated therapies. The outcomes of the treatment were divided into four categories: complete response (i.e. when calcifications resolved in 100%), partial response (any response other than 0 and 100%), no response, and unknown response (in case of patients who were lost to follow-up for any reason).

### Risk of bias

The risk of bias was assessed with the Joanna Briggs Institute’s critical appraisal tools [16]. The overall bias was ranked as low when > 75% of questions were answered “Yes”, high if the prevalence was < 50%, and moderate otherwise.

### Data analysis

We extracted data of individual patients from each study and summarized the outcomes of all evaluated treatment modalities with the descriptive statistics. We reported medians with ranges for continuous variables and frequencies for categorical variables. Missing data were managed by mean imputation.

## Results

### Study selection

**Fig. 1:**
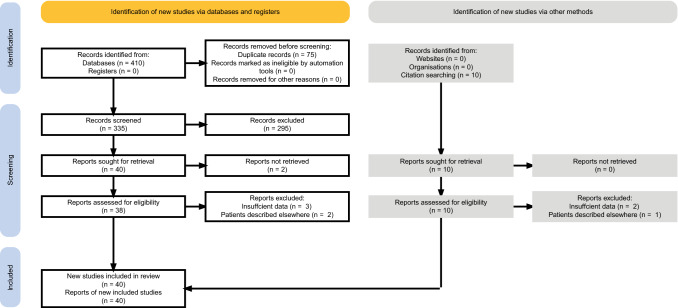
PRISMA flowchart illustrating the process of data screening

A total of 410 articles were retrieved from the search. Additionally, ten more studies were added after the manual screening of the reference sections [10, 17–25]. Eight articles were excluded at the eligibility stage due to reporting patients described in other included studies [20, 26, 27] or insufficient data [24, 25, 28–30]. In the selection process, 40 records met the eligibility criteria and were included in this systematic review (Fig. 1). Details of the included studies are specified in Table 1.

**Table 1:** Characteristics of the studies included in the systematic review

First author (publication year)	No of cases	Underlying disease	Location of lesion(s)	Maximal diameter of the largest lesion [cm]	Treatment frequency, range	Median (range) treatment duration (months)	Median (range) time to the first clinical improvement (months)	Outcomes^a^	Median (range) follow-up (months)
Topical sodium thiosulfate									
Abbott [59]	1	IV calcium infusion extravasation	Hand	4.0	Twice a day	10 weeks	1	CR	6
von Hodenberg [32]	6	SSc × 6	Fingers	0.4	Once a day	8 (range 2–13)	N/R	2/6 CR, 4/6 PR	N/R
Bhari [42]	1	EB	Hands, feet	0.5	Twice a day	3	N/R	PR	3
Ma [31]	28	SSc × 15, overlap × 4, UCTD × 4, SLE, CLE, JDM, RA, JRA	Hands, feet, lower extremities, upper extremities	N/R	Twice or three times a day	Mean 3.9 (range 0.3–24)	Mean 4 (range 0.5–11)	2/28 CR, 17/28 PR, 7/28 NR, 2/28 UR	N/R
Karthik [40]	1	Werner syndrome	Lower extremity	13.0	N/R	9	5	PR	15
Tajalli [39]	1	CREST	Finger	0.3	Three times a day	3	2	CR pain free	> 36
Topham [38]	1	JDM	Lower extremity	N/R	Three times a week	2	N/R	PR	N/R
García-García [41]	1	IV calcium infusion extravasation	Upper extremity	N/R	Twice a day	6	3	CR pain free regained mobility	N/R
Jost [18]^b^	3	TC × 2, HHS	Buttock, lower extremity, upper extremity	20	Once a day	13 (range 4–13.4)	4 (range 2–7)	1/3 CR, 2/3 PR pain reduction × 1	14 (range 7–14)
Urretavizcaya [33]	2	IV calcium infusion extravasation × 2	Upper extremity	N/R	N/R	N/R	N/R	PR regained mobility	N/R
Pagnini [48]	1	JDM	Lower extremities, upper extremities	N/R	Once a day	9	N/R	PR	N/R
Perez-Moreno [46]	1	IV calcium infusion extravasation	Upper extremity	N/R	N/R	N/R	2 weeks	CR pain free regained mobility	4
Wolf [47]	1	SLE	Lower extremities	12.5	Twice a week	N/R	6	PR pain reduction	10
Intralesional sodium thiosulfate									
López-Sundh [43]	1	SSc	Axillae	3.0	Once a month	3	N/R	CR pain reduction	N/R
Olesen [17]	29	SSc × 26, overlap × 2, DM	N/R	65.0	Once a week	4 weeks	N/R	13/29 CR, 13/29 PR, 3/29 NR	(12–16 weeks)
Winter [22]	5	DM × 2, SSc × 2, morphea	N/R	2.0	Once a month	3	N/R	1/5 CR, 3/5 NR, 1/5 UR	3
Tonial [21]	7	SSc × 6, DM	Earlobe, fingers, hands, lower extremities, upper extremities	N/R	Once or twice a month	1.5 (range 1–3.5)	N/R	NR 4/5 pain reduction	N/R
Goossens [10]	2	DM, TC	Buttock, upper extremity	20.4	Once or twice a week	16.5 (12–21)	2.5 (2–3)	2/2 PR 2/2 pain reduction	16.5 (12–21)
Gunasekera [36]	1	SLE	Abdomen, lower extremities, upper extremity	N/R	Two or three sessions	N/A	N/R	CR	16
Oh [19]	1	JDM	Lower extremity	2.4	Once a month	6	4	PR pain reduction	N/R
Baumgartner-Nielsen [4]	6	SSc × 5, NSF	Buttocks, fingers, lower extremities, upper extremity	8.5	Single session or once a week	1 week (3–4 weeks)^c^	1	2/6 CR, 4/6 PR 6/6 pain reduction	12 weeks
Smith [37]	1	DM	Finger	0.4	Once a week	2 weeks	1 week	CR pain free	3 weeks
Co_2_ laser									
Cannarozzo [35]	5	scrotal calcinosis	Scrotum	N/R	Single session	N/A	N/R	5/5 CR	7 years (range 2–10)
Aristazabal [44]	1	milia-like calcinosis	Forehead	N/R	Single session	N/A	N/R	PR	6
Weig [49]	1	SCN	Finger	> 0.6	Single session	N/A	N/R	CR	18
Zarate [45]	1	JDM	Fingers, hand, upper extremity	1.0	Single session	N/A	N/R	PR pain free	6
Kutlubay [50]	1	trauma	Lower extremity	0.4	Single session	N/A	2 weeks	CR	3
Joo [51]	1	SCN	Earlobe	2.0	Single session	N/A	N/R	CR	5 weeks
Chamberlain [52]	1	CREST	Fingers	N/R	Single session	N/A	6 weeks	PR	> 36
Paek [23]	1	SCN	Eyelid	N/R	Single session	N/A	N/R	CR	15
Bottomley [13]	6	SSc × 6	Fingers	N/R	Single session	N/A	(8–16 weeks)	12/21 lesions CR, 5/21 lesions PR, 4/21 lesions NR 3/6 pain free 2/6 pain reduction	> 10
Er:YAG laser									
Meissner [53]	1	CREST	Buttocks	< 2.0	Single session	N/A	2 weeks	CR	14 weeks
Diode laser									
Wollina [54]	1	scrotal calcinosis	Scrotum	N/R	Single session	N/A	N/R	CR	N/R
Picosecond laser followed by CO_2_ laser									
Abrouk [55]	1	DM	Hips	N/R	Three sessions	N/R	N/R	CR	N/R
ESWL									
Delgado-Márquez [57]	1	Overlap	Lower extremity	8.0	Once every 2 weeks	N/R	N/R	PR pain reduction	N/R
Sultan-Bichat [5]	8	CVI × 4, SSc × 3, DM	Fingers, lower extremities	N/R	Once every 3 weeks	9 weeks	N/R	1/8 CR, 4/8 PR, 2/8 NR, 1/8 UR 3/8 pain free 2/8 pain reduction	6
Chan [56]	1	JDM	Lower extremity, upper extremity	1.5	Single session	N/A	N/R	NR pain reduction	> 6
Sparsa [11]	1	CREST	Lower extremities	< 20	Once a month	3	2 weeks	PR	N/R
ESWL followed by intralesional sodium thiosulfate									
Pavlov-Dolijanovic [58]	1	SSc	Lower extremities	5.0	Once a week	22 weeks	N/R	PR pain reduction	N/R
Picosecond laser followed by topical sodium thiosulfate									
Eleryan [34]	2	JDM, DM	Buttocks, lower extremity	5.0	Twice a week/month	6	N/R	2/2 PR 1/2 pain reduction	N/R

*CLE* cutaneous lupus erythematosus, *CO*_*2*_ carbon dioxide, *CR* complete response, *CVI* chronic venous insufficiency, *DM* dermatomyositis, *EB* epidermolysis bullosa, *Er:YAG* erbium-doped yttrium aluminium garnet, *ESWL* extracorporeal shockwave lithotripsy, *HHS* hyperphosphatemia hyperostosis syndrome, *IV* intravenous, *JDM* juvenile dermatomyositis, *N/A* not applicable, *n of patients* number of patients, *NSF* nephrogenic systemic fibrosis, *N/R* not reported, *PR* partial response, *RA* rheumatoid arthritis, *JRA* juvenile rheumatoid arthritis, *SCN* subepidermal calcified nodule, *SLE* systemic lupus erythematosus, *SSc* systemic sclerosis, *STS* sodium thiosulfate, *TC* tumoral calcinosis, *UR* unknown response

^a^Pain and mobility improvement were assessed in patients that experienced pain and reduced range of motion before local treatment

^b^One patient in this study was previously described by Ratsimbazafy et al.[20]

^c^Calculated only for the patients who underwent multiple sessions

### Quality assessment

Included articles consisted of one prospective double-blind placebo-controlled pilot study [22], 7 case series [4, 5, 10, 13, 17, 18, 21, 31–35], and 27 case reports [36–44]. Five conference abstracts were included [19, 21, 33, 45, 46]. In two studies only, a control group was present and defined as another lesion in the same patient [22, 34]. The overall risk of bias was ranked as low in 10 studies, moderate in 19, and high in 11 (Supplementary Content).

### Patients’ characteristics

A total of 136 patients with CC were evaluated in this systematic review. The youngest was a neonate while the eldest was 91 years old. Man to woman ratio was 21:90 (sex of 25 patients was not reported). Majority of patients had an underlying cause of CC, most commonly an autoimmune connective tissue disease. Predominantly, patients were diagnosed with systemic sclerosis (*n* = 71), dermatomyositis or juvenile dermatomyositis (*n* = 16), and overlap syndrome (*n* = 7). The duration of CC symptoms ranged from a few days (in case of extravasation) to 30 years (in systemic sclerosis). All lesions were clinically assessed by a physician before and after the treatment. Calcifications were observed throughout the body (extremities, trunk, pelvic area, earlobes, and eyelids) and were often characterised by the presence of ulceration, infection, pain, and limited mobility.

### Treatment modalities

Top-STS was prescribed as a compounded drug due to the absence of commercial products on the market. Formulation base was prepared from cold cream (*n *= 17), zinc ointment (*n *= 27), gel base (*n *= 1, in a patient allergic to zinc), petrolatum (*n *= 1), and lotion (*n *= 1). Compresses soaked with sodium thiosulfate (STS) were used in one case alternately with acetic acid dressings and steroid ointment applied around the wound edges [47]. In some studies, the treated areas were left under occlusion. Concentration of STS in the formulas was 10% (*n *= 18), 20% (*n *= 1), or 25% (*n *= 30). In one case, the concentration used was gradually increased from 3 to 10% over 2 weeks [48], while in another, the concentration was increased from 25 to 50% after 1 year of treatment [31]. In one study, reported STS doses corresponded to 370 mg and 833 mg per day [18].

The use of IL-STS was reported at the following concentrations: 0.1 mg/ml (*n *= 7), 40 mg/ml (*n *= 5), 150 mg/ml (*n *= 31), and 250 mg/ml (*n *= 7). Doses of STS ranged from 9.27 mg to 3000 mg per appointment and depended on the lesion sizes and patients’ tolerance as the injections might be painful despite anaesthesia [9, 10].

In patients treated with CO_2_, Er:YAG, and diode lasers only one session was required [13, 23, 35, 35, 44, 49–54]. One patient who underwent therapy with a picosecond laser followed by a CO_2_ laser required three sessions [55]. Among the CO_2_ lasers, the ultrapulse variant was most commonly reported. Configuration of the laser settings was vast and unfeasible to summarise.

Some studies indicated the use of ESWL in both focalised and non-focalised modes with 2500–3000 shock waves and the power of 896–929 V per treatment (energy of 0.1–0.3 mJ/mm^2^) [5, 56, 57]. Calcifications could be located by a fluoroscopic amplification device coupled to the lithotripter [5].

Pavlov-Dolijanovic et al. [58] used combined therapy consisting of 6 sessions of ESWL at weekly intervals followed by a 6-week break and continued with 4 weekly IL-STS injections (150 mg/ml). Eleryan et al. [34] reported the use of 6 and 7 monthly sessions of picosecond laser followed by application of 4 mL of 5% top-STS.

### Outcomes of the treatment

Improvement was achieved in 81% (39/48) of cases treated with top-STS and 74% (39/53) with IL-STS. In those who underwent ESWL and laser therapies, response to treatment was observed in 64% (7/11) and 71% (15/21) of cases, respectively. The patients who were subject to the ESWL therapy combined with IL-STS and picosecond laser followed by top-STS had partial remission. Detailed information about the outcomes is provided in Table 2.

**Table 2:** Summary of local, non-systemic, and minimally invasive treatment options for calcinosis cutis

Treatment modality	*n*	Maximal diameter of the largest lesion (cm)	Treatment frequency, range	Median (range) treatment duration (months)^a^	Median (range) time to the first clinical improvement (months)^a^	Response rates, %, *n/N*	Adverse events (number of patients)
STS							
Topical	48	13	Twice a week to three times a day	3.9 (0.3–24)	4.0 (0.5–11)	Complete, 19, 9/48	Transient pruritus (1), recurrence (1), skin irritation (2), zinc ointment allergy (1)
						Partial, 63, 30/48	
						None, 6, 3/48	
						Unknown, 13, 6/48	
Intralesional	53	65	Single session to once a month	1.0 (0.5–21)	1.0 (0.25–4)	Complete, 36, 19/53	Infection (5), injection pain (6^b^), blistering (1), skin discoloration (N/R)
						Partial, 38, 20/53	
						None, 25, 13/53	
						Unknown, 2, 1/53	
Laser							
CO_2_	18	2.0	One to three sessions	N/A^c^	N/A^c^	Complete, 57, 12/21	Infection (2), recurrence (2), scaring or hyperkeratosis (10), hypopigmentation (2), itching or burning (2)
Er:YAG	1	< 2.0				Partial, 14, 3/21	
Diode	1	N/R				Unknown, 29, 6/21^d^	
Picosecond + CO_2_	1	N/R					
ESWL	11	< 20.0	Single session to twice a month	2.25 (1–2.25)	N/A^c^	Complete, 9, 1/11	Transient fever (1)
						Partial, 55, 6/11	
						None, 27, 3/11	
						Unknown, 9, 1/11	
Combined							
ESWL + intralesional STS	1	5.0	Once a week	5.5	N/R	Partial, 100, 1/1	Crater-like defects (N/R)
Picosecond + topical STS	2	5.0	Twice a week to twice a month	6.0	N/R	Partial, 100, 2/2	None

*Er:YAG* erbium-doped yttrium aluminium garnet, *ESWL* extracorporeal shockwave lithotripsy, *N/A* not applicable, *N/R* not reported, *STS* sodium thiosulfate

^a^Missing data were managed by available case analysis and mean imputation was used when a mean was known for a variable of interest but exact values were not reported for individual cases from a single study

^b^Olesen et al. [17] also reported injection pain, however, without an exact number of cases experiencing it

^c^A group of patients was too small

^d^Bottomley et al. [13] reported outcomes as for particular lesions instead of describing overall response in each patient

Patients who experienced partial or complete reduction in pain were reported mainly in the ESWL (pain-free, 3/10; reduced pain, 4/10) and laser (pain-free, 4/9; reduced pain, 2/9) subgroups. Data regarding pain control were poorly reported for STS. Median pain reduction measured in the VAS scale was 3 (range 0–9) in six patients treated with ESWL, 1 (range 0–5) in five patients managed with IL-STS, and 8 in the patient managed with ESWL followed by IL-STS.

### Perioperative management

Intradermal or topical anaesthesia was utilised during IL-STS [10], CO_2_ [13, 35, 44, 52], and Er:YAG laser procedures [53]. Some patients were prescribed with nonsteroidal anti-inflammatory drugs or paracetamol after the procedure. Postoperative management included an ointment or cream of healing and soothing properties [55], often combined with antimicrobials such as chlorhexidine [50], mupirocin [13, 52], fusidic acid [35, 44], and povidone-iodine [53].

### Adverse effects

Infection was recognised in 9% (5/53) of patients after IL-STS [4, 10, 17] and in 11% (2/18) of cases managed with CO_2_ laser (no infectious complications were reported for other types of lasers) [13]. Infections were managed with antibiotics [10, 47]. Injection-associated pain was transient and occurred in over 11% of patients receiving IL-STS. Either scaring or hyperkeratosis was noticed in 56% (10/18) of patients after CO_2_ laser treatment [13, 35, 50]. Furthermore, three recurrences within a few months were observed after top-STS and CO_2_ laser therapies [13, 18]. In one such case, the calcifications recurred after a trauma [18]. One case of transient fever was the only adverse reaction observed after ESWL [56]. Adverse effects of each treatment modality are specified in Table 2.

## Discussion

This review summarises the current evidence about the local and minimally invasive therapies for calcinosis cutis. A single treatment modality lead to a partial or complete remission in the majority of patients (from 64 to 81%). Combined treatment was reported only for three patients to date. Small calcifications (the largest reported length of 4 cm [59]) can be completely treated locally, although recalcitrant CC may require systemic therapy. The results of local therapies for idiopathic and iatrogenic CC are especially promising (87% complete response, 13/15), potentially owing to the absence of further triggering factors for formation of calcifications. Available evidence speaks for safety of local methods owing to manageable mild side effects and absence of severe adverse reactions.

### STS

STS is a substance known for its use in cyanide poisoning, cisplatin toxicity [18], and calciphylaxis [60]. Its use for CC is justified, as STS is presumed to act as a chelating agent that increases calcium solubility up to 100,000 times [4, 59, 61]. Taking into account the supposed mechanism of action, its impact on bone mineralisation should be investigated, especially in the paediatric population [18]. Additionally, anti-inflammatory (hydrogen sulfide production), vasodilative (nitric oxide synthase regeneration), and antioxidative (reduction of free radicals) properties of STS may support tissue healing in CC [18, 41, 59]. Intravenous STS may be used for CC, however, this route of administration is considered impractical [10, 30] and have moderate to life-threatening adverse events, such as headaches, vomiting, metabolic acidosis, hypotension, and hypernatremia [4, 10, 18, 41].

Top-STS shows promising outcomes (81% response rate) and seems to have the least systemic influence among the local treatments for CC. Nonetheless, patient compliance is required owing to longer duration of treatment compared to other modalities (Table 2). Apparently, topical route and drug self-application might facilitate the long-term adherence in comparison to injections and oral treatment [18, 32]. Top-STS may be investigated as potential treatment for lesions disqualified from conventional surgery (i.e. disseminated and extensive lesions and these located in anatomically critical sites [5, 17, 62]). Some patients with a history of unsuccessful therapy with top-STS respond to IL-STS [10, 30], laser [45], and ESWL [57]. Interestingly, a similar compound, sodium metabisulfite in concentration of 25%, was suggested as an effective alternative to STS [63, 64].

STS absorption into the bloodstream after intralesional injection seems to be of low level as the untreated lesions of patients receiving this therapy remained unchanged and no alterations in anion gap, eGFR, calcium, bicarbonate, and chloride serum levels were observed [10]. High complete response rates (of 36%) observed after IL-STS may result from increased concentrations of the active substance in the lesion site when compared to other routes of administration [10]. IL-STS may facilitate the treatment of CC in patients with thickened skin in systemic sclerosis, which is an obstacle for an accurate surgical removal [13]. Transient injection-associated pain was a common adverse effect (in over 11%). Healing lesions may be prone to infection (9%, emerging mainly from the skin microbiome) due to softened calcifications [10] and injury caused by the injection. Some patients experienced less pain after completed therapy (median VAS scale reduction of 1, range 0–5), despite unsuccessful size reduction of calcifications [21]. Twelve patients reported by Winter et al. [22] (with control lesions) and Tonial et al. [21] demonstrated no significant change in size after 3–8 IL-STS injections, although low doses and long injection intervals could disadvantage the treatment efficacy in these studies.

### Laser treatment

Laser therapy is considered less invasive, faster, and more convenient than conventional surgery for both patients and clinicians. A single session is usually enough for the complete removal of small calcifications (up to 2 cm long [51]). No recurrences were observed within the follow-up, in contrast to conventional surgery, when the soft tissue injury from procedure may induce the formation of new lesions [2, 12, 14, 17]. Spontaneous extrusion of the chalky exudate (expelling the softened calcium deposits) after laser therapy and ESWL is considered as a good sign of the healing process [5, 55]. In comparison to curettage, laser treatment helps to achieve a good haemostasis by closing capillaries, which ensures a clear operating field [13, 52]. Excellent aesthetic results with a minimal scar may be achieved by the use of precise laser vaporisation [14, 52]. Lower risk of thermal damage is associated with Er:YAG laser as it has ten times greater absorption than CO_2_ laser and encourages wound healing [53]. In one study, picosecond laser was used as an assisted drug delivery for top-STS, achieving partial regression of calcifications [34]. Skin discoloration or scarring was a common side effect (56%). Infections and recurrences were also noted in several cases (11% for both events).

### ESWL

ESWL is a minimally invasive procedure with a low risk of adverse effects. It is widely used for urolithiasis and calcific tendonitis [5, 11, 56, 57], or adjuvantly for wound healing [65]. This procedure seems the most effective for ulcerated or radiopaque (calcium oxalate or dihydrate) calcinosis on small areas, regardless the location and underlying disease [5]. Its efficacy is lower for thick and extensive plaques or calcifications showing radio-transparency (cystine or uric acid) [5]. ESWL destroys the structure of calcifications without their mechanical extraction [11] and for that reason a prior use of ESWL may facilitate a surgical extraction of calcifications [5]. ESWL is effective for larger calcifications (up to 20 cm long [11]), however, it leads only to a partial reduction in size (in 55%, while complete remission is noted only in about 7%) [56, 57]. ESWL shows a satisfactory analgesic effect (median VAS scale reduction of 3, range 0–9), possibly owing to the fragmentation of calcified lesions that compress nerves and tissues [5]. This method could be, therefore, considered as a pain relief and palliative treatment for CC, especially as an opioid-sparing strategy for extensive and painful lesions [56]. Assessment of the true efficacy of ESWL combined with IL-STS is necessary as these methods could possibly complement each other [58]. A limitation of ESWL is the decreased accessibility to some lesions in individuals with limited mobility as positioning of patients was primarily designed for urologic conditions [5].

### Limitations

Studies reporting the treatment of CC present a low level of evidence due to the lack of high quality and adequately powered randomized controlled trials and the absence of head-to-head studies, which results from the low prevalence of CC [41, 59]. Thus, this review is based on weak evidence. Despite that, we believe it may have some value for clinicians looking for guidance in the management of this rare disease.

## Conclusion

This study provides an overview of local and minimally invasive therapies that could become the life-changing treatment for selected patients with CC, with potential better safety and non-inferior efficacy to systemic drugs and surgical excision. Insufficient evidence is currently available to provide strong recommendations regarding these treatment modalities. For pain reduction, ESWL and IL-STS may be considered. Convenient self-application at home is an advantage of top-STS. Lasers are highly effective for solitary microcalcifications. Disadvantages of IL-STS include transient pain during injection. Clinical trials are required for further confirmation of these findings.

## Supplementary Information

Below is the link to the electronic supplementary material.

Supplementary file1 (PDF 239 KB)
