# Supplementary material for: Local, non-systemic, and minimally invasive therapies for calcinosis cutis: a systematic review
Source: Arch Dermatol Res. 2021 Jun 24;314(6):515–25. doi: 10.1007/s00403-021-02264-5 (PMC9232439; doi:10.1007/s00403-021-02264-5)
Supplement: Supplementary file 1 — Supplementary file1 (PDF 239 KB) [file 403_2021_2264_MOESM1_ESM.pdf]

Local, non-systemic, and minimally invasive therapies for calcinosis cutis: a systematic review

Journal: Archives of Dermatological Research

Authors (ORCID iDs): Joanna Nowaczyk (0000-0001-7438-3559)[a], Michał Zawistowski (0000-0002-5173-6283)[a], Piotr Fiedor, MD, PhD, Prof.[a]

[a]Department of General and Transplantation Surgery, Medical University of Warsaw, Warsaw, Poland

Corresponding author: Piotr Fiedor, MD, PhD, Prof., Department of General and Transplantation Surgery, Medical University of Warsaw, Nowogrodzka 59, 02-006 Warsaw, Poland, email: piotrfiedor@wp.pl

Supplementary Content

Table S1. Search queries used to collect the data

Database	Search query	Filters	Date of last search	Number of records
**Pubmed**	((Calcinosis[MeSH Terms] AND Skin[MeSH Terms]) OR (calci* cutis)) AND ("sodium thiosulfate"[Supplementary Concept] OR Lithotripsy[MeSH Terms] OR "Laser Therapy"[MeSH Terms])	none	March 14, 2021	153
**Embase**	'skin calcification'/exp AND ('sodium thiosulfate'/exp OR 'extracorporeal shock wave lithotripsy'/exp OR 'laser'/exp)	none	March 14, 2021	128
**Web of Science**	((skin calcification) AND ((sodium thiosulfate) OR (extracorporeal shock wave lithotripsy) OR ESWL OR laser))	none	March 14, 2021	129

Table S2. Risk of bias assessment with the Joanna Briggs Institute Critical Appraisal tools.

Study	Study design	Q1[a]	Q2	Q3	Q4	Q5	Q6	Q7	Q8	Q9	Q10	Q11	Q12	Q13	Overall risk[b]
Topical sodium thiosulfate															
Abott et al. (2020)	Case report	✓	✗	✓	✓	✓	✓	?	✓						Moderate
von Hodenberg et al. (2020)	Case series	✓	✓	✓	✓	✓	✗	?	?	✗	NA				High
Bhari and Bharti (2019)	Case report	✓	✗	✓	?	?	✗	✗	✓						High
Ma et al. (2019)	Case series	✓	✓	✓	✓	✓	✓	✓	✓	✓	✓				Low
Karthik et al. (2019)	Case report	✓	✗	✓	✓	?	?	✗	✗						High
Tajalli et al. (2019)	Case report	✓	✗	✓	✓	✓	✓	✓	✓						Low
Topham et al. (2019)	Case report	✓	✗	✓	✓	✗	✗	✗	✓						High
García-García et al. (2017)	Case report	✓	✗	✓	✓	✓	✓	✓	✓						Low
Jost et al. (2016)	Case series	✓	✓	✓	✓	✓	✓	✓	✓	✓	NA				Low
Urretavizcaya et al. (2016)	Case series	?	?	✓	?	?	?	?	?	?	NA				High
Pagnini et al. (2014)	Case report	✓	✗	✓	✓	?	✓	✗	✓						Moderate
Perez-Moreno et al. (2014)	Case report	✓	✗	?	?	?	?	✓	✓						High
Wolf et al. (2008)	Case report	✓	✗	✓	✓	✓	✓	?	✓						Moderate
Intralesional sodium thiosulfate															
López-Sundh et al. (2020)	Case report	?	✓	✓	✓	✓	✓	✓	✓						Low
Olesen and Fage (2020)	Case series	✓	✓	?	✓	✗	✓	?	?	✓	✗				Moderate
Winter et al. (2020)	Double-blind, placebo-controlled pilot study	✓	✓	✓	✓	✓	✓	✓	✗	✓	✓	✓	✓	✓	Low
Tonial et al. (2018)	Case series	✓	✓	?	?	?	?	?	✓	✓	✓				Moderate
Goossens et al. (2017)	Case series	✗	✓	✓	?	?	✓	✓	✓	✓	NA				Moderate
Gunasekera et al. (2017)	Case report	✓	✗	✓	✓	✓	✓	✓	✓						Low
Oh et al. (2017)	Case report	✓	✗	?	?	?	?	✗	✗						High
Baumgartner-Nielsen and Olesen (2016)	Case series	✗	✓	?	✓	✓	?	✓	✓	?	NA				Moderate
Smith (2013)	Case report	✓	✗	✓	✓	✓	✓	✓	✓						Low
CO_2 laser															
Cannarozzo et al. (2020)	Case series	✓	✓	?	?	?	?	✗	✓	✓	NA				High
Aristazabal et al. (2019)	Case report	?	✓	✓	✓	✓	✓	✗	✓						Low
Weig et al. (2019)	Case report	✓	✗	✓	✓	✓	✓	?	✓						Moderate
Zarate et al. (2017)	Case report	✓	✗	✓	✓	✓	?	?	✓						Moderate
Kutlubay et al. et al. (2014)	Case report	✓	✗	✓	✓	✓	✓	✓	✓						Low
Joo et al. (2004)	Case report	✓	✗	✓	✓	✗	?	✗	✓						Moderate
Chamberlain and Walker (2003)	Case report	✓	✗	?	?	✓	?	✓	✓						Moderate
Paek et al. (1996)	Case report	✓	✗	✓	✓	?	?	✗	✓						Moderate

Bottomley et al. (1996)	Case series	✓	?	?	?	?	✓	✓	✓	✓	NA	Moderate
Er:YAG laser												
Meissner et al. (2010)	Case report	✗	✗	✗	✗	✗	✗	✗	✓			High
Diode laser												
Wollina (2012)	Case report	✓	✗	?	✗	✓	?	✗	✓			High
Picosecond + CO_2 laser												
Abrouk et al. (2020)	Case report	✓	✗	?	✗	✓	✗	✗	?			High
Extracorporeal shock wave lithotripsy												
Delgado-Márquez et al. (2014)	Case report	✓	✗	✓	✓	?	✓	?	✓			Moderate
Sultan-Bichat et al. (2011)	Case series	✓	✓	?	✓	?	✓	✓	✓	✓	NA	Moderate
Chan and Li (2005)	Case report	✓	✗	✓	✓	✓	?	✓	✓			Moderate
Sparsa et al. (2005)	Case report	✓	✗	?	✓	?	?	✓	✓			Moderate
Extracorporeal shock wave lithotripsy followed by intralesional sodium thiosulfate												
Pavlov-Dolijanovic et al. (2018)	Case report	✓	✗	✓	✓	?	?	✓	✓			Moderate
Picosecond laser followed by topical sodium thiosulfate												
Eleryan et al. (2019)	Case series	✓	?	✓	✗	?	?	✓	✓	✓	NA	Moderate

Abbreviations: *Q*, question.

Legend: ✓, Yes; ✗, No; ?, Unclear; NA, not applicable.

[a]Questions based on the JBI risk assessment checklists (see: https://joannabriggs.org/critical-appraisal-tools) for respective study designs.

[b]Overall risk of bias judgement was ranked as low in case >75% of questions were answered “Yes”, high if the prevalence was <50%, and moderate otherwise.
